# Computational Studies of Marine Toxins Targeting Ion Channels 

**DOI:** 10.3390/md11030848

**Published:** 2013-03-13

**Authors:** M. Harunur Rashid, Somayeh Mahdavi, Serdar Kuyucak

**Affiliations:** School of Physics, University of Sydney, New South Wales 2006, Australia; E-Mails: harun@physics.usyd.edu.au (M.H.R.); mahdavi@physics.usyd.edu.au (S.M.)

**Keywords:** conotoxins, ShK toxin, ion channels, docking, molecular dynamics, potential of mean force, free energy perturbation

## Abstract

Toxins from marine animals offer novel drug leads for treatment of diseases involving ion channels. Computational methods could be very helpful in this endeavour in several ways, e.g., (i) constructing accurate models of the channel-toxin complexes using docking and molecular dynamics (MD) simulations; (ii) determining the binding free energies of toxins from umbrella sampling MD simulations; (iii) predicting the effect of mutations from free energy MD simulations. Using these methods, one can design new analogs of toxins with improved affinity and selectivity properties. Here we present a review of the computational methods and discuss their applications to marine toxins targeting potassium and sodium channels. Detailed examples from the potassium channel toxins—ShK from sea anemone and *κ*-conotoxin PVIIA—are provided to demonstrate capabilities of the computational methods to give accurate descriptions of the channel-toxin complexes and the energetics of their binding. An example is also given from sodium channel toxins (*µ*-conotoxin GIIIA) to illustrate the differences between the toxin binding modes in potassium and sodium channels.

## 1. Introduction

Voltage-gated ion channels play key roles in electrical signalling in cells. They function much like transistors—A change in the membrane potential opens the channel gate and allows passive diffusion of a selected type of ions such as Na^+^,K^+^, or Ca^2+^ across the cell membrane [1]. Dysfunction of ion channels due to mutations in the channel protein or environmental effects are associated with numerous diseases [2]. Thus ion channels are important targets for therapeutic drugs, and there is an ongoing interest in the pharmaceutical industry to find channel blockers with high affinity and specificity. Many toxins from marine animals bind to specific ion channels with high affinity [3,4], and therefore provide natural leads for drug development [5,6,7,8,9,10]. 

Once a toxin is identified as a potential drug lead for a target ion channel, more work needs to be done to improve its affinity and selectivity for the target. This is essential to reduce the dosage and avoid side effects that may arise from binding of the drug to unintended proteins. This may be achieved in the lab by creating analogs of the toxin through mutations of selected residues, and testing their affinity for various proteins. Such a trial and error approach could be very time consuming and success is not guaranteed. Provided the structures of the target proteins are available—either from X-ray diffraction or via homology modelling—one can alternatively use computational methods to construct accurate models for the channel-toxin complexes and predict the effect of the mutations in silico. Advances in crystallization of membrane proteins and computer hardware/software in the last fifteen years have made such a computational approach to drug design a distinct possibility. 

Availability of a crystal structure of an ion channel is essential for computational studies of toxin binding. Channel models constructed in the absence of a crystal structure are not reliable enough to use in atomistic simulations. The first crystal structure of a bacterial potassium channel (KcsA) was determined in 1998 [11], followed by many others [12]. Of particular importance for toxin binding studies was the solution of the mammalian voltage-gated potassium channel Kv1.2 [13], which has enabled construction of homology models for other Kv1 channels. Sodium channels were relatively harder to crystallize. The first crystal structure for a bacterial voltage-gated sodium channel appeared only recently [14], and a mammalian one is yet to be solved. Unlike potassium channels where the pore domains of bacterial and mammalian channels are very similar, there are substantial differences between the two classes in sodium channels. Thus constructing homology models of the mammalian Nav1 channels from the bacterial crystal structure will be a more challenging task. As yet, there are no crystal structures for the calcium channels and the ligand-gated ion channels, which explains the current focus of the computational studies on the potassium and sodium channels. 

The most important progress in computer hardware was the introduction of the cluster architecture and parallel computing, which brought supercomputing power to masses. This was an essential breakthrough because an accurate description of structure and dynamics of a complex system requires an atomic-level treatment via molecular dynamics (MD) simulations and sufficient sampling of the simulation system. Routine simulation of a protein system consisting of ∼ 10^5^ atoms in the microsecond range would not have been feasible without the high-performance computing power afforded by the clusters. On the software front, MD programs and their associated force fields such as AMBER [15], CHARMM [16], and GROMACS [17] have been continuously improved since their inception. Used in combination with a docking program, MD simulations have the ability to produce accurate models of protein-ligand complexes [18]. Similarly, one can perform free energy simulations to predict the absolute free energy of binding for a given complex, and predict the change in the binding free energy due to a mutation in the complex near chemical accuracy [19,20,21,22]. 

Here we present a review of the computational methods used in construction of channel-toxin complexes, and calculation of absolute and relative binding free energies in such complexes. Because application of these methods to protein-peptide complexes are relatively new, we provide detailed examples from the potassium channel toxins ShK and *κ*-conotoxin PVIIA. Computational investigation of sodium channel toxins is just starting. Nevertheless, we give an example from *µ*-conotoxin GIIIA to illustrate how the binding modes in sodium channels differ from those in potassium channels. 

## 2. Computational Methods

There are only a few crystal structures for complexes of membrane proteins. Thus the first step in a computational study of toxin binding to ion channels is the construction of complex structures. Here accuracy of the model structure is of utmost importance because without an accurate complex model, free energy calculations in the next step have no chance of succeeding. Accordingly, we first discuss the computational methods used in structure prediction followed by the free energy methods. 

### 2.1. Complex Structure Prediction from Docking and MD Simulations

In order to find the structure of a channel-toxin complex, one first needs the individual structures of the channel and the toxin. Those of toxins can be determined using NMR in a straightforward manner, and many toxin structures are available from the protein data bank. Structures of channel proteins are determined from X-ray crystallography, and because it is much harder to crystallize membrane proteins, not many channel structures are available. Hence, one has to rely on homology modeling in most cases. The situation is relatively better in potassium channels where several crystal structures exist [12], including the mammalian voltage-gated potassium channel Kv1.2 [13]. Thus one can construct homology models of other Kv1 channels relatively easily, although it is still very important to validate such model channels using available functional data. In sodium channels, the crystal structure of a bacterial channel was determined recently [14], which has opened the way for homology modelling of the mammalian Nav1 channels. Due to substantial differences between the bacterial and mammalian sodium channels, proper validation of the homology models is even more important in this case. 

Two main methods for prediction of protein-ligand complexes are docking and MD simulations. Docking programs allow fast screening of many ligands for a given target [23,24], but their accuracy is limited [25]. Conversely, MD simulations provide accurate representation of the protein-ligand interactions but they are too slow to predict the complex structure from scratch. Combination of the two methods, where the initial binding poses predicted by a docking method are refined in subsequent MD simulations, offers the most practical approach for finding complex structures. Initial applications of this approach to small ligands (< 50 atoms) using common docking programs such as AUTODOCK [26] and ZDOCK [27] produced promising results [18,28,29,30]. Its feasibility for larger and more flexible peptide ligands was first shown for the KcsA potassium channel-charybdotoxin complex [31,32]. The structure of this complex was determined from NMR experiments [33], so it could be used for testing the accuracy of the docking plus MD approach and the effectiveness of the docking programs [32]. Using a more sophisticated docking program such as HADDOCK [34,35], which allowed flexibility and ensemble docking, was found to give superior results compared with rigid docking programs [28,32]. 

Docking programs rank the complex poses according to their energy score. Inspection of the top 10 or 20 poses is usually sufficient to make a decision on an initial pose. A systematic study of potassium channel-toxin complexes using HADDOCK has shown that a consensus complex is obtained in most cases [32]. We assume this to be the case in the following, but if more than one pose is found from docking, each pose needs to be refined with MD. After an initial configuration for the complex structure is chosen from docking, one needs to prepare the simulation system for refinement with MD. There are well-established protocols for this purpose [36,37], which can be used for the complex model as well. Typically, the complex model is embedded in a lipid bilayer and solvated with a salt solution using the VMD program [38]. The resulting system is gradually relaxed in MD simulations until it reaches equilibrium. Care needs to be exercised to ensure that all the disulfide and hydrogen bonds in the complex structure are preserved during relaxation. Once the system is well equilibrated in MD simulations, a production run is performed for analysis of the binding mode. Snapshots of the complex system can be used to get a visual picture of the binding mode. A more quantitative description can be obtained by calculating the average pair distances for the strongly interacting residues. Charge interactions, where the N–O distance between the charged residues is less than 3 Å, and hydrophobic interactions involving the benzyl groups provide the strongest couplings (> 2 kcal/mol). Hydrogen bonds and charge interactions at larger distances are of intermediate strength (1–2 kcal/mol). Where available, comparison of the alanine scanning mutagenesis data with the predictions of the complex model provides the best validation for the proposed model. If no mutation data are available, one has to rely on binding free energies, which is discussed in the next section. 

There are several MD programs that one can use for refinement of the complex. In the examples discussed here, the NAMD code [39] is used with the CHARMM force field [16] including the CMAP correction for the dihedral terms [40]. The NpT ensemble is the most suitable one for MD simulations of biomolecules and has been adapted in most MD studies. The temperature and pressure can be maintained at the standard values of 300 K and 1 atm using temperature and pressure coupling. Employment of the periodic boundary conditions avoids artefacts arising from using small boundary boxes and also facilitates computation of the long-range electrostatic interactions. Neglecting the long-range electrostatic interactions using cut-off distances causes errors and is not recommended. The current practice is to include them using the particle-mesh Ewald algorithm. The short-range Lennard-Jones interactions can be switched off within a distance of 10–13.5 Å without causing errors. A typical time step used in the MD simulations is 2 fs. Using longer time steps results in accumulation of systematic errors while shorter ones require more computing time and are not used unless extreme accuracy is desired. For details of the basic formalism of MD simulations, we refer to the monographs [41,42,43]. A recent review of MD simulations of membrane proteins can be found in [44]. 

### 2.2. Free Energy Calculations

Binding constants of toxins are routinely measured and readily available for most channel-toxin complexes. Thus, in the absence of mutation data, they provide the only means for validating a complex model. This, in turn, requires an accurate calculation of the binding constant. There are two classes of methods that can be used for this purpose: (i) path independent alchemical perturbation methods, where the ligand is simultaneously destroyed in the binding pocket while created in bulk; and (ii) path dependent potential of mean force (PMF) methods, where the ligand is physically pulled from the binding pocket to bulk [19,20]. Here the PMF gives the continuous free energy profile of the toxin along the chosen reaction coordinate. The first method is computationally cheaper but its application to peptide toxins—which are flexible and have many charged residues—suffers from sampling problems [45]. Thus until the problems in applying the perturbation method to charged-flexible ligands are resolved, the PMF approach will remain the method of choice. 

Formally, the binding constant is determined from the 3-D integral of the PMF of the ligand, *W* (r) 

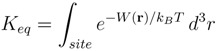
(1)
where it is assumed that *W* = 0 in bulk. Because computation of a 3-D PMF is not feasible, one invokes a 1-D approximation and determines the PMF along the reaction coordinate, which is the channel axis for a channel-toxin complex. Taking the channel axis along the z axis, the binding constant is given by 

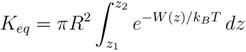
(2)
where *z*_1_ and *z*_2_ refer to the toxin’s center of mass (COM) coordinates at the binding site and in the bulk, respectively. The factor *πR*^2^ measures the average cross-sectional area of the binding pocket as explored by the COM of the toxin, and the radius *R* is determined from the transverse fluctuations of the COM of the toxin in the binding pocket. The accuracy of this approximation has been checked for ions by independent free energy perturbation calculations [46]. Because toxins are much heavier, their transverse fluctuations along the *z* axis are further suppressed compared with ions, so the 1-D approximation should work even better for toxins. The absolute binding free energy is determined from the binding constant using 

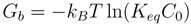
(3)
where *C*_0_ is the standard concentration of 1 mol/liter (*i.e*., 1/*C*_0_ = 1661 Å^3^). 

The most common method used in PMF calculations is the umbrella sampling MD simulations, where one introduces harmonic potentials to enhance sampling of the ligand at high-energy points [47]. For convenience, umbrella potentials are introduced at regular intervals (e.g., 0.5 Å) along the reaction coordinate. To generate the simulation windows, a harmonic force with *k* in the range of 20–40 kcal/mol/ Å^2^ is applied to the COM of the toxin backbone, which is pulled at a constant speed of 5 Å/ns over 0.5 Å. After each pulling step, the toxin is equilibrated at the window position by applying the same harmonic restraint for 200 ps to relax the effect of steering on the environment. Initially windows are generated for up to 15 Å starting from the binding pocket, which are extended further if necessary (*i.e*., until the PMF becomes flat). If insufficient overlap occurs between the adjoining windows (typically less than 5%) due to a local potential barrier, an extra window is included in the middle of the two windows. The COM coordinates of the toxin, measured with respect to the COM of the channel, are collected during the umbrella sampling MD simulations. The PMF is obtained using the weighted histogram analysis method [48], which unbiases the COM coordinates in each window and combines them in an optimal way. Convergence of the PMF is the sole criterion on how long one should run each window, which can be studied using block data analysis. Typically PMF obtained from individual blocks of data first monotonically decreases and then fluctuates around a base line. In the first phase, the system is still equilibrating and the data should be discarded. Fluctuations in the second phase indicate that equilibration has been reached, so the final PMF should be constructed using the production data from this part. 

An alternative method for constructing the PMF of a ligand is to use Jarzynski’s equation [49] in steered MD simulations [50]. Due to its simplicity, this method has become quite popular in recent years. However, detailed comparisons with the umbrella sampling method indicate that its application to biomolecules suffers from sampling problems and the convergence of PMFs is too slow to be useful in practice [51,52]. 

The PMF method was first applied to the KcsA potassium channel-charybdotoxin complex, where the structure was known, hence providing an important test case [31]. In this study, a large discrepancy was found in the calculated absolute binding free energy, which was caused by the distortion of the toxin during umbrella sampling simulations. In a follow-up study, conformational restraints were used to prevent the deformation of the toxin, which enabled calculation of the absolute binding free energy within chemical accuracy of 1 kcal/mol [53]. Since then, the PMF method has been used in several computational studies of toxin binding to ion channels [54,55,56,57,58,59,60,61]. As long as a validated complex structure was employed in the PMF calculations, the absolute binding free energy was obtained within chemical accuracy in all cases. 

Improving the affinity and selectivity of a drug lead via mutations poses a less taxing computational problem, as one is interested in the change in the binding free energy due to a mutation. Provided the binding mode is not altered by the mutation, this is most efficiently calculated using the alchemical transformation methods such as free energy perturbation (FEP) and thermodynamic integration (TI) [62]. In both methods, one introduces a hybrid Hamiltonian, *H*(λ) = (1 − λ)*H*_0_ + λ*H*_1_, where *H*_0_ represents the Hamiltonian in the initial state (wild type ligand) and *H*_1_ in the final state (mutant ligand). The alchemical transformation is performed by changing the parameter λ from 0 to 1 in small steps, which ensures that the change in the free energy in each step is small enough to enable sufficient sampling of the system in a reasonable time frame. In the FEP method, the interval [0,1] is divided into *n* subintervals with {λ*i*,*i* =1,*n* − 1}, and for each subinterval the free energy difference is calculated from the ensemble average 

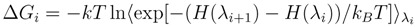
(4)
The free energy difference between the initial and final states is obtained from the sum, Δ*G* = Σ_*i*_ Δ*G_i_*. The number of subintervals (windows) is chosen such that the free energy change at each step does not exceed 2 kcal/mol, otherwise the method may lose its validity. For mutations of involving charged residues, this requires over hundred windows if uniform subintervals are used. Using exponentially spaced subintervals instead, one could reduce the number of windows substantially. 

In the TI method, the ensemble average of the derivative, *∂H(λ)/∂λ*, is obtained at several *λ* values, and the free energy difference is calculated from the integral, 

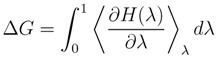
(5)
The TI method is especially advantageous for mutations involving charged residues, because Gaussian quadrature allows evaluation of the integral using a small number of windows, which can be sampled for longer times to check convergence of the results. A seven-point quadrature was found to be adequate in previous applications of the TI method [46,63]. In both methods, it is important to perform the backward calculation to check for hysteresis effects. If the difference between the forward and backward results is much larger than 1 kcal/mol, the calculated free energies are not reliable, most likely due to insufficient sampling of the system. 

Mutation of a charged residue to a neutral one is a more challenging problem and requires additional considerations to avoid sampling problems. Firstly, one needs to preserve the net charge in the system during the calculations. This can be achieved by performing the following thermodynamic cycle: While a charged residue on the toxin in the binding site is mutated to a neutral one, the reverse transformation is simultaneously applied to a mutant toxin in bulk. Secondly, the Coulomb and Lennard-Jones (LJ) interactions need to be handled separately, which can be implemented by introducing uncharged residues as intermediate steps. For example, the free energy change due to a Lys to Ala (*K* → *A*) mutation can be expressed as

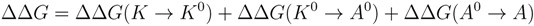
(6)
The first term represents the discharging of the side chain of a Lys residue on the bound toxin while the reverse process is performed on a toxin in bulk with an uncharged Lys side chain. In the second term, the uncharged Lys side chain is transformed to an uncharged Ala side chain while the reverse is performed on the bulk toxin. Finally, the third term corresponds to charging of the Ala side chain in binding site while the one in bulk is discharged. Each of these contributions to the free energy difference can be calculated using the FEP or TI methods. 

## 3. Potassium Channel Toxins

Due to earlier availability of crystal structures, potassium channels have been the main focus in computational investigations of marine toxins targeting ion channels. Homology models of the voltage-gated potassium channels Kv1 can be constructed using the crystal structure of the Kv1.2 channel in a straightforward manner. As shown in Table 1, there is one-to-one correspondence among the pore domain residues, thus the models can be simply constructed using the mutator plugin in the VMD software [38]. The only important point to note in modeling of the Kv1 channels is the residue following the DM signature in the extended region, which corresponds to T449 in Shaker, Y379 in Kv1.1, V381 in Kv1.2, and H404 in Kv1.3. In both Shaker and Kv1.3 the side chain of this residue makes a cross-link with the side chain of the neighboring D residue (*i.e*., T449–D447 in Shaker and H404–D402 in Kv1.3). There is no such cross-linking in Kv1.1 because the Y379 side chain is too bulky to fit around the filter. If the models of Shaker and Kv1.3 are not properly relaxed in MD simulations, these cross-links could break, resulting in a wrong model of the channel, where the T449/H404 side chains project out of the pore. The protruding T449/H404 side chains interfere with the binding of toxins and prevent their correct docking to Shaker [64] and Kv1.3 channels [54,55]. 

**Table 1:** Alignment of the Shaker and rat Kv1 channel sequences depicting the differences in the turret and extended regions.

		Turret	Pore helix	Filter	Extended region	
Shaker	418	EAGSENSFFK	SIPDAFWWAVVTMT	TVGYG	DMT PVGVW	454
Kv1.1	348	EAEEAESHFS	SIPDAFWWAVVSMT	TVGYG	DMYPV T I G	384
Kv1.2	350	EADERDSQFP	SIPDAFWWAVVSMT	TVGYG	DMVPT T I G	386
Kv1.3	373	EADDPSSGFN	SIPDAFWWAVVTMT	TVGYG	DMHPV T I G	409

**Figure 1:**
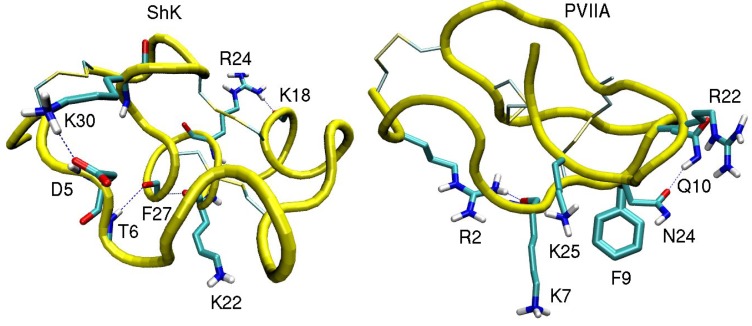
NMR structures of the ShK toxin and *κ*-conotoxin PVIIA oriented with the pore inserting lysine pointing downward. In ShK, there are three disulfide bonds (C3–C35, C12–C28, and C17–C32), and three other bonds (D5–K30, K18–R24 and T6–F27), which make the structure very stable. In PVIIA, there are three disulfide bonds (C1–C16, C8–C20, and C15–C26), and two other bonds (R2–K7 and Q10–N24).

In this section we discuss binding of ShK toxin from sea anemone and *κ*-conotoxin PVIIA to Kv1 potassium channels in some detail to show that computational methods can provide accurate descriptions of the channel-toxin complexes and the energetics of their binding. These examples are chosen because of the availability of the alanine scanning mutagenesis data, which allow a direct validation of the proposed complex models. NMR structures of ShK [65] and PVIIA [66] are shown in Figure 1. 

### 3.1. ShK Toxin

The Kv1.3 channel is an established target for the treatment of autoimmune diseases, such as multiple sclerosis and rheumatoid arthritis [67,68]. ShK toxin from the sea anemone Stichodactyla helianthus [69,70] binds to the Kv1.3 channel with an IC5_0_ of 11 pM [71], hence it provides an excellent lead for development of an immunosuppressant drug. However, ShK also binds to other Kv channels with high affinity, in particular Kv1.1 with an IC5_0_ of 16 pM [71]. Lack of specificity for Kv1.3 prevents the use of ShK as a therapeutic drug. Therefore, there is an intense effort for developing analogs of ShK that have improved selectivity for Kv1.3 over Kv1.1 and other potassium channels [9,10]. Many ShK analogs have been created for this purpose, and some had the required selectivity, e.g., ShK-Dap22 [71], ShK-186 [72], and ShK-192 [73]. However, the use of non-natural amino acids or adducts in these analogs has limited their potential for development as drugs. For example, the phosphorylated Tyr residue in ShK-186 is prone to hydrolysis. Therefore, developing Kv1.3 selective analogs of ShK from natural amino acids is more desirable. 

**Figure 2:**
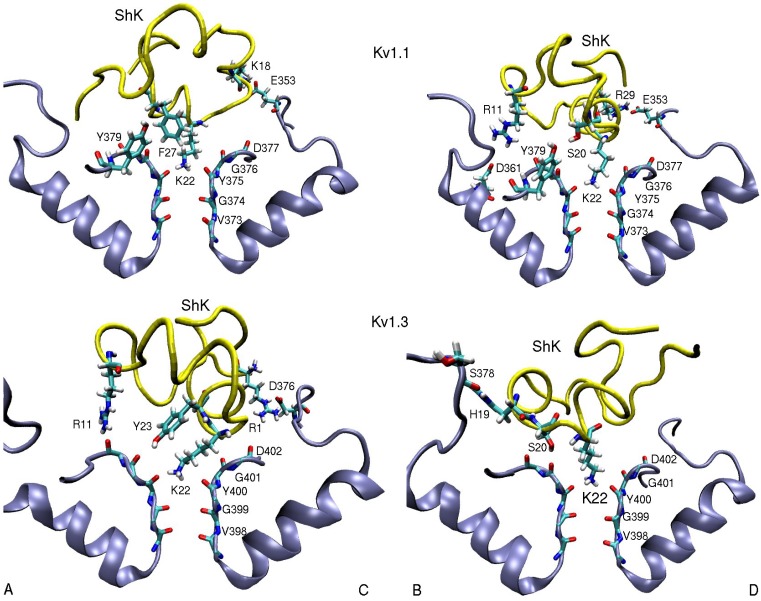
Snapshots of the Kv1.1–ShK and Kv1.3–ShK complexes showing the strongly interacting residues. Only those involved in the binding are indicated explicitly. In order to show all the residues involved in binding, two views of the complex are presented depicting the channel monomers A and C, and B and D separately.

**Figure 3:**
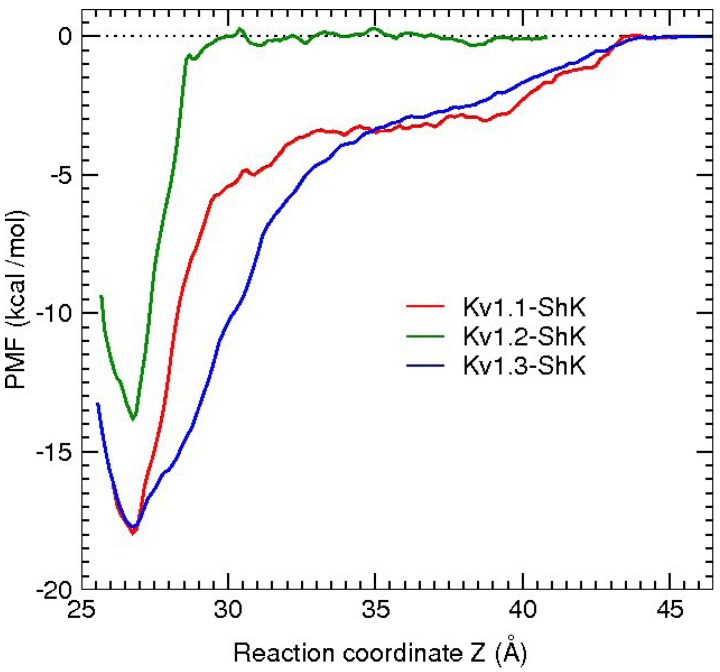
Comparison of the PMFs obtained from the umbrella sampling simulations for the unbinding of ShK from the Kv1.1, Kv1.2, and Kv1.3 channels.

An essential first step in searching for selective analogs using structure-based drug design methods is to construct accurate models of the protein-ligand complexes involved in the selectivity problem. This has been recently achieved for the Kv1.x–ShK complexes using docking and molecular dynamics (MD) simulations [59]. Snapshots of the equilibrated Kv1.1–ShK and Kv1.3–ShK complexes are shown in Figure 2. A more quantitative description of the strongly interacting residues is provided in Table 2, which shows the average atom-atom distances obtained from 5 ns of unrestrained MD simulations for each complex. Comparison of the results for the Kv1.3–ShK complex with the alanine scanning mutagenesis data [74] shows that the complex model accounts for all the strongly interacting residues identified in the experiment. The only exception is the mutation R24A, which is an allosteric effect. R24 is not involved in the binding but its mutation to alanine breaks the R24–K18 bond (see Figure 1), which changes the shape of the ShK mutant and its binding mode. Further evidence for the validity of the complex models are provided by the binding free energies, which are obtained from the integration of the PMFs shown in Figure 3. In all three cases, the PMF calculations of the binding free energies have reproduced the experimental values within chemical accuracy [59]. 

**Table 2:** List of the strongly interacting residues in the ShK–Kv1.x complexes. The average atom-atom distances obtained from MD simulations are given in units of Å (standard deviations are not listed as they are less than 0.4 Å). The N–O distances are shown for the charge interactions and the closest C–C distance for the hydrophobic ones. Bare C, N, and O refer to the backbone atoms and the subscripted ones refer to the side chain atoms. The monomer identity is given at the end of the residue number.

ShK–Kv1.1	dist.	ShK–Kv1.2	dist.	ShK–Kv1.3	dist.
				R1(N_1_)–D376(O_1_)C	4.5
R11(N_2_)–D361(O_2_)B	5.5	S10(O_H_)–D353(O_2_)B	2.8	R11(N_2_)–D402(O)A	3.5
K18(N_1_)–E353(O_2_)C	2.7			H19(N)–S378(O)B	3.0
S20(O_H_)–Y379(O_H_)B	3.0	M21(N)–D379(O)D	3.1	S20(O_H_)–G401(O)B	2.7
		M21(C_*γ*_)–V381(C_*γ*2_)D	3.8	M21(C_*ε*_)–V406(C_*γ*1_)B	4.7
K22(N_1_)–Y375(O)	2.7	K22(N_1_)–Y377(O)	2.7	K22(N_1_)–Y400(O)	2.7
				Y23(O_H_)–G401(O)A	3.5
F27(C_*ε*2_)–Y379(C_*ε*1_)A	3.6			F27(C_*ε*1_)–H404-C_*γ*_)C	3.6
R29(N_2_)–E353(O_2_)D	2.5	R29(N_1_)–D355(O_1_)A	2.7	R29(N_1_)–D376(O_1_)C	10.2

Comparison of the binding modes of the Kv1.1–ShK and Kv1.3–ShK complexes (Figure 2 and Table 2) gives valuable hints for improving the selectivity of ShK for Kv1.3 over Kv1.1. The side chains of K18 and R29 in ShK are strongly coupled to the glutamate side chains in Kv1.1 via ionic bonds but they do not exhibit any interactions with the Kv1.3 residues. Thus mutation of the either residue in ShK to alanine could lead to a substantial selectivity gain for Kv1.3 over Kv1.1. To test this hypothesis, binding of the K18A and R29A mutants of ShK to the Kv1 channels have been studied employing the same protocols used for ShK. The R29A mutation was found to alter the binding mode in both Kv1.1 and Kv1.3, and therefore was not pursued. The K18A mutation did preserve the binding mode in both channels, and hence the effect of the K18A mutation on the selectivity free energy could be calculated using the FEP and TI methods. Because the validity of these methods for charge mutations was not well established, the binding free energies of ShK-K18A were also calculated as a test of FEP and TI. All three methods gave similar results and predicted that the K18A mutation would improve the Kv1.3/Kv1.1 selectivity free energy by about 2 kcal/mol, which was confirmed in subsequent experiments. 

An amidated analogue of ShK (ShK-K-amide) has been known to improve the Kv1.3/Kv1.1 selectivity but the mechanism of this selectivity was not clear. A similar computational study performed for ShK-K-amide showed that this mutation had negligible effect on its binding to Kv1.3 but grossly altered its binding mode to Kv1.1 [60]. The binding free energies of ShK-K-amide calculated from the respective PMFs again reproduced the experimental values within chemical accuracy, increasing the confidence in the accuracy of the computational methods. 

### 3.2. *κ*-conotoxin PVIIA

The drosophila Shaker (Shaker) channel is the first voltage-gated potassium channel to be cloned, and therefore it is often used as a role model in investigation of the structure and function of potassium channels. Similarly, *κ*-PVIIA is the first conotoxin found to block Shaker [75], and thus it has attracted a great deal of attention. The binding mode of *κ*-PVIIA to Shaker was studied via mutagenesis experiments [76,77], and it has also been used in many functional studies of Shaker channels [78,79,80,81]. Structure and mutagenesis data indicate that *κ*-PVIIA binds to Shaker via a functional dyad consisting of a pore-inserting lysine (K7) and a hydrophobic residue (F9), similar to the binding mode of other toxin blockers of potassium channels [82,83]. In an earlier computational study of the Shaker-*κ*-PVIIA complex [64], the functional dyad was reproduced but the other important interactions identified in the mutagenesis experiments [77] were not. As stressed earlier, this was due to incorrect modelling of the pore region of Shaker. 

**Figure 4:**
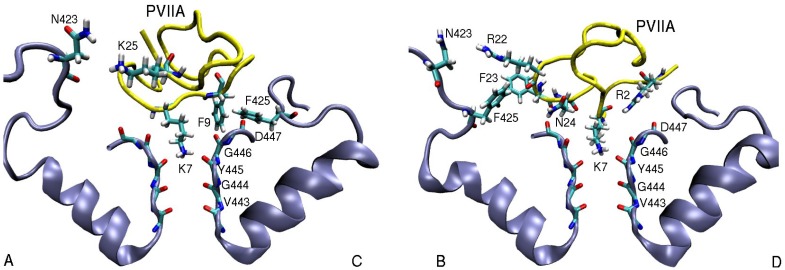
Snapshots of the Shaker-*κ*-PVIIA complex showing the important residues involved in binding. The monomers A and C and B and D are shown separately for clarity.

In a more recent study of *κ*-PVIIA binding to Shaker and Kv1 channels [61], a correct model of Shaker was used and the results obtained from the Shaker-*κ*-PVIIA complex model were in general agreement with the experiments. Similar protocols as in ShK were employed in creating the complex model. Snapshots of the Shaker-*κ*-PVIIA complex is shown in Figure 4. The pairs of residues involved in binding and the average distances between the pairs of atoms—obtained from the MD simulations of the complex—are given in Table 3. Comparison of the pairs of residues in Table 3 with the alanine scanning mutagenesis data [77] shows that all strongly interacting residues identified in the experiment have been accounted for in the complex structure. The binding energies of *κ*-PVIIA to Shaker and Kv1.2 have also been reproduced within chemical accuracy, providing further validation for the complex models. 

**Table 3:** Similar to Table 2 but for the *κ*-PVIIA–Kv1 complexes. Benz refers to the COM of the benzyl group.

*κ*-PVIIA–Shaker	dist.	*κ*-PVIIA–Kv1.1	dist.	*κ*-PVIIA–Kv1.2	dist.
R2(N_1_)–D447(O)D	3.9	R2(N_2_)–D377(O)A	5.0	R2(N_2_)–D379(O)D	3.7
Q6(N_1_)–D447(O)A	4.7	Q6(N_1_)–D377(O)A	5.1	Q6(N_1_)–D379(O)A	3.2
K7(N_1_)–Y445(O)	2.7	K7(N_1_)–Y375(O)	2.8	K7(N_1_)–Y377(O)	2.7
F9(C_*ζ*_)–T449(C_*γ*2_)C	4.4	F9(C_*δ*2_)–Y379(C_*є*2_)C	3.9	F9(C_*є*1_)–V381(C_*γ*1_)C	4.7
F9(Benz)–F425(C_*ζ*_)C	3.6				
R22(N_1_)–N423(O_1_)B	6.2	R22(N_2_)–E351(O_2_)B	3.0	R22(N_2_)–D355(O_1_)B	3.5
F23(Benz)–F425(C_*ζ*_)B	5.0				
N24(N_1_)–D447(O)B	3.0	N24(N_1_)–D377(O)C	3.8	N24(N_1_)–D379(O)C	2.9
K25(N_1_)–D447(O_1_)B	6.1	K25(N_1_)–Y379(O_1_)A	4.7	K25(N_1_)–D379(O)B	2.9
K25(N_1_)–N423(O_1_)A	5.0				

**Figure 5:**
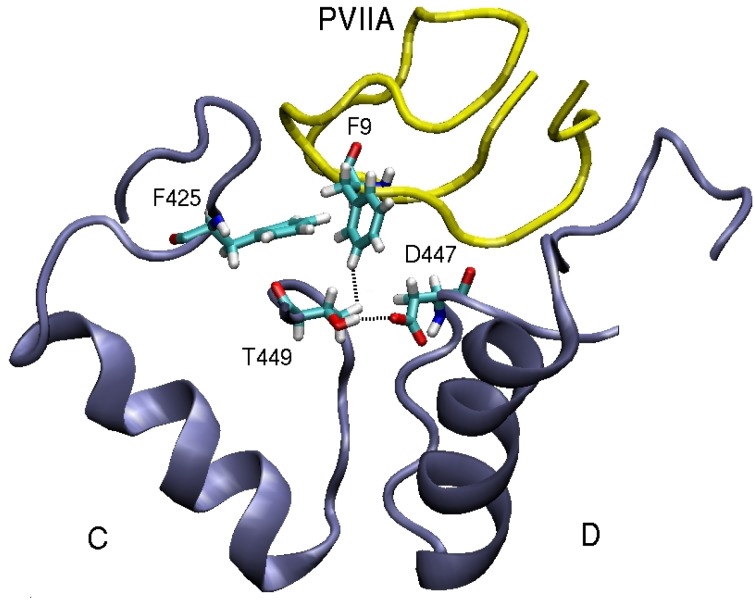
An alternative view of the Shaker-*κ*-PVIIA complex that demonstrates the importance of the T449–D447 cross-linking in preserving the strong F9–F425 hydrophobic interaction.

In contrast to Shaker, Kv1 channels are found to be insensitive to *κ*-PVIIA [75,76]. This finding can be understood by comparing the binding modes of *κ*-PVIIA to Shaker and Kv1 channels. Inspection of Table 3 shows that the hydrophobic interactions are missing in the Kv1-*κ*-PVIIA complexes. A detailed picture of the binding mode in Shaker (Figure 5) helps to explain why. The T449–D447 cross-linking keeps the threonine side chain away from the strongly coupled F9–F425 pair. Indeed the T449Y mutation breaks this cross-linking, which disrupts F9–F425 coupling and renders Shaker insensitive to *κ*-PVIIA. In a similar fashion, lack of cross-linking in the corresponding residues in Kv1.1 and Kv1.2 prevents the formation of hydrophobic interactions, making them insensitive to *κ*-PVIIA. 

## 4. Sodium Channel Toxins

**Figure 6:**
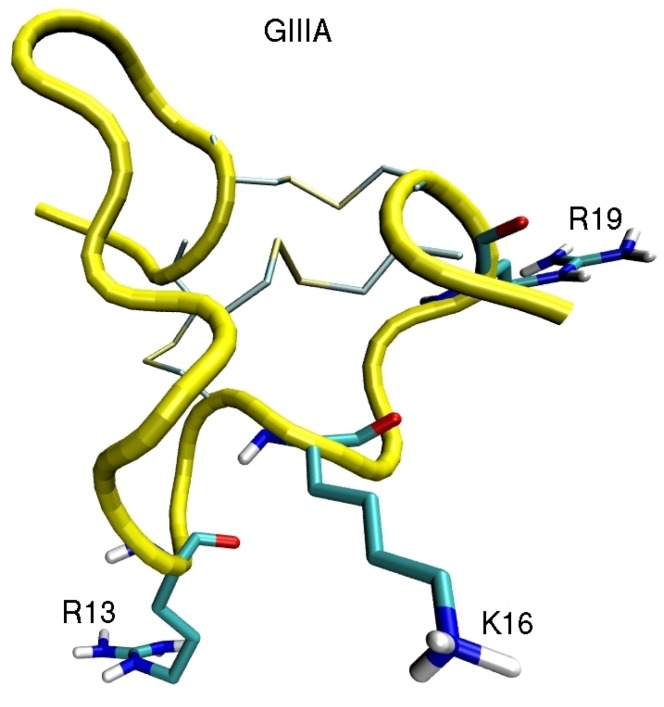
NMR structure of *µ*-GIIIA [98] showing the important residues involved in binding to Nav1.4.

Computational investigation of sodium channels has just started after the first crystal structure for the bacterial channel NavAb was solved [14]. Two more bacterial Nav channels have been solved since then [84,85]. There have been some earlier model studies of Nav-toxin complexes based on potassium channels and experimental data [86,87,88], but their accuracy is limited. So far, binding of *µ* conotoxin to NavAb [56] and scorpion *β*-toxins to the voltage sensor [58] have been investigated. Due to substantial differences between the bacterial and mammalian Nav channels, homology modelling of the pore domain requires careful validation [89]. Here we briefly discuss binding of *µ*-conotoxin GIIIA to Nav1.4 to illustrate the differences between the toxin binding modes in potassium and sodium channels (see Figure 6 for the NMR structure of *µ*-GIIIA). *µ*-GIIIA is the first conotoxin found to block Nav channels [90], and numerous functional studies of its binding to Nav1.4 have been performed [91,92,93,94,95,96,97]. Thus there is a wealth of mutation data to validate the Nav1.4-*µ*-GIIIA complex models. 

**Figure 7:**
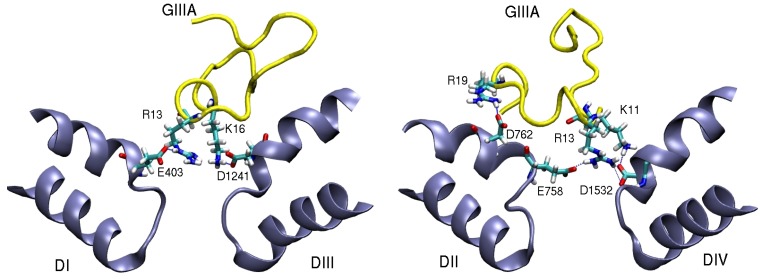
Snapshots of the Nav1.4-*µ*-GIIIA complex showing the important residues involved in binding. The domains D1–DIII and DII–DIV are shown separately for clarity.

A homology model of the pore domain of Nav1.4 was created by aligning the DEKA residues with the corresponding EEEE residues in NavAb. The Nav1.4-*µ*-GIIIA complex was created using the same procedures as in potassium channel studies. Snapshots of the complex model (Figure 7) shows that *µ*-GIIIA interacts mainly with the outer ring EEDD residues but has no coupling to the inner ring DEKA residues. R13 makes multiple connections with residues in three domains of Nav1.4 (E403, E758, and D1532), and it is clearly the pore blocking residue, consistent with the mutation experiments [91,92]. K16 is also involved in two interactions (E758 and D1241), again in agreement with the mutation data [87,95,97]. The third important coupling is provided by the K11–D1532 interaction. The proposed model of the Nav1.4-*µ*-GIIIA complex gives a satisfactory account of the available mutation data. The binding free energy of *µ*-GIIIA is determined from the PMF calculations and has been found to agree with the experimental value within chemical accuracy. Thus the Nav1.4-*µ*-GIIIA complex has been well validated and could be used as a template in constructing homology models for the pore domain of other Nav1 channels, which are highly homologous. 

**Figure 8:**
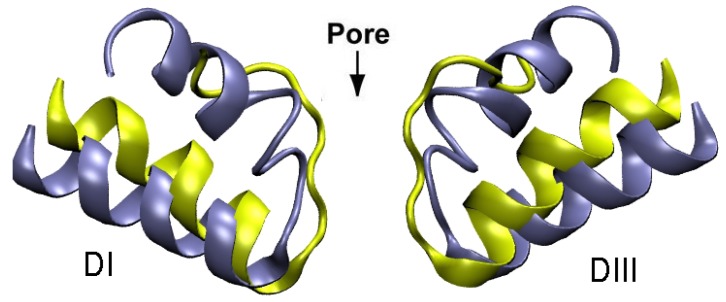
Comparison of the pore domains of Kv1.2 (yellow) and NavAb (blue) channels involved in toxin binding.

It is of interest to compare the pore domains of the potassium and sodium channels, and point out the differences in toxin binding modes arising from structural constraints. As shown in Figure 8, the selectivity filter in potassium channels is very narrow, long, and has a highly negative potential. This potential attracts a Lys residue (but not the larger Arg) into the filter, which completely blocks the channel. In sodium channels, the selectivity filter is wider and shorter, but the DEKA locus has a smaller negative potential. As a result, toxins preferentially interact with the EEDD residues in the outer ring. Because the outer ring is even wider, only an Arg residue interacting with several domains can completely block the channel—a Lys residue provides only a partial block. The wider opening in sodium channels prevents formation of a tight binding mode observed in many potassium channel-toxin complexes leading to pM affinities. Thus a crucial issue in designing drug leads for sodium channels from toxins is how to increase their relatively low affinities. 

## 5. Conclusions

Rapid advances in computer hardware and software in the last decade has enabled accurate description of the structure and energetics of protein-ligand complexes. As shown in the present work, the accuracy of the computational methods now extends to description of channel-toxin complexes. This opens new avenues for design of drugs from natural sources such as marine toxins. Affinity and selectivity properties of a toxin for a given channel target can be improved by making rational choices for mutations from accurate complex models, and then performing free energy calculations to find the free energy change associated with the mutation. While very good results have been obtained in the examples discussed here, more tests need to be performed to check the predictive power of the computational methods and increase the confidence in them. This is especially so in sodium channels, whose crystal structure has been solved only recently, and there are only a few computational studies of ligand binding to sodium channels as yet. It is expected that there will be a lot of activity in simulation of ligand binding to sodium channels in near future, which will bring our understanding of ligand binding to a level similar to that in potassium channels. 

The computational methods have an enormous potential for rational drug design from toxins targeting ion channels, but lack of crystal structures for many types of ion channels is hindering progress. Solution of the structures of channel proteins is the main bottleneck at present and more effort should go in this direction. For example, the ligand-gated ion channels such as nicotinic acetylcholine receptor are even more important therapeutic targets than the voltage-gated ion channels [2], and there are many families of toxins that bind to these channels with high affinity and selectivity. Thus solution of the structures of these channels would enable application of the computational methods for the purpose of designing novel therapeutic agents from the toxin blockers of these channels. 
